# The importance of surprising findings in psychology

**DOI:** 10.3389/fpsyg.2013.00173

**Published:** 2013-04-09

**Authors:** David Trafimow

**Affiliations:** Department of Psychology, New Mexico State UniversityLas Cruces, NM, USA

There can be little doubt that surprising findings have been emphasized in psychology. To publish in top empirical psychology journals, it is important for reviewers to feel that the findings have a low prior probability; i.e., that the findings were improbable. My goal is to show that there are different ways in which a finding can be surprising and that the usual way does not further the science of psychology.

Trafimow (2003) used the famous theorem by Bayes to show that if a surprising prediction is confirmed by experiment, it creates more of a difference between the prior and posterior probability of a theory than if an unsurprising prediction is confirmed by experiment. Intuitively, it seems inescapable that if a theory makes surprising predictions that actually come out, this is a more impressive accomplishment than if a theory does not make surprising predictions. The history of science is replete with examples of extremely important experiments that successfully confirmed surprising predictions made from theories such as Einstein's theory of relativity.

To understand my argument, it is important to fully appreciate the notion of *auxiliary assumptions*. As philosophers have noted, theory-based predictions rely on (1) the theory and (2) auxiliary assumptions that provide the link between non-observational terms in theories and observational terms in predictions that derive from theories. For example, in the middle part of the nineteenth century, many scientists felt that it was impossible to test theories about the chemical composition of the stars because there was no way to travel to a star to procure star material for chemical analysis. However, subsequent advances in spectroscopy made it possible to connect light spectra to the combinations of elements that generate them. Thus, the advances in spectroscopy provided auxiliary assumptions that could be used to test theories about the chemical composition of stars.

The reason for harping on auxiliary assumptions, and particularly the auxiliary assumptions that determine methodology, is that their existence suggests two dimensions regarding how surprising theory-based predictions are. One dimension would pertain to how surprising the theory is whereas the other dimension would pertain to how surprising the auxiliary assumptions are. By considering the extreme poles along these two dimensions, it is easy to imagine four combinations (but only the first two are important). First, suppose that the theory is surprising and the auxiliary assumptions are obvious, so that the surprising predictions are surprising because of the theory. In that case, there would be high confidence that the predictions actually test the theory. Therefore, if the predictions work out, the theory would be considered to have passed a strong test, and there would be an impressive difference between its prior and posterior plausibility. In this case, empirical confirmation of surprising predictions constitutes a non-trivial advance for psychology.

For example, consider the widely accepted theory, often called “correspondence bias” or the “fundamental attribution error,” that there is a general tendency for people to not pay attention to information about situations (e.g., see Jones, 1990 for a review). Trafimow (1998) suggested a contradictory theory that suggests that people pay a great deal of attention to situations but that researchers have not detected this because their methods involved asking for judgments about traits, which unintentionally primed participants to not use information about the situation. To avoid this problem, Trafimow used a person memory paradigm whereby participants were led to believe that a target person was kind or unkind either at work or at home, the participants were subsequently presented with congruent, and incongruent behaviors ostensibly performed at work or at home, and later were asked to recall all of the items. It had been well-established by previous research that people tend to recall information that is incongruent with their attributions about people better than congruent information. The empirical issue at hand, then, was whether participants would recall incongruent behaviors better than the congruent ones regardless of whether they were performed at work or at home, or whether participants would exhibit a situation-specific incongruity effect. Trafimow obtained the surprising finding of a situation-specific incongruity effect that contradicted the widely accepted theory that people habitually commit a fundamental attribution error, and supported that people pay much attention to situations after all. The finding was not surprising at the empirical level, as few would doubt that in the paradigm Trafimow used, a general expectancy should lead to a general incongruity effect whereas a situation-specific expectancy should lead to a situation-specific incongruity effect. The surprise was at the theoretical level—that participants could so easily be induced to form situation-specific expectancies given the dominant assumption of a strong tendency for people to not consider situations.

Second, let us consider what I believe to be the most usual case in psychology, where the theory is not particularly surprising but one or more auxiliary assumptions are. In more typical language, what makes many psychology predictions surprising is the prior unlikelihood that the methodology actually would work, so that when it works, reviewers are impressed. It is easy to imagine a colleague saying to the experimenter, “Wow, you got that to work!” But does this way of being surprising actually advance psychology? Consider that if the theory is not surprising, it has a pretty good prior probability of being true. For a silly example, my “theory” (“empirical generalization” might be a more accurate description) that 6:00 news shows have a tendency to come on at approximately 6:00 would be judged by most people to be likely to be true even without any formal experiments. So if the theory is already judged to be likely to be true, there is little room for even the cleverest set of experiments to make an impressive difference from prior to posterior probability. At this point, I ask the reader to consider the latest issue of his or her favorite empirical psychology journal, and judge what proportion of the articles in the journal contained a surprising theory, or whether the surprise was in the auxiliary assumptions. The same question, put another way, is for the reader to consider whether the surprise was at the level of the theory or at the level of the experimental hypothesis. Assuming that the reader would agree that the surprise usually was not in the theory itself, but rather that surprising auxiliary assumptions are what led to the surprising predictions, did the article cause a large change between subjective prior and posterior judgments of how likely the theory was to be true? If not, I would suggest that not much was gained that was of impressive scientific value.

Consider an example. Petty and Cacioppo (1984) proposed the obvious theory that sometimes people put more effort into processing persuasive messages and sometimes they put less effort into doing so. After putting participants into a mindset where they would put more or less effort into processing persuasive messages, Petty and Cacioppo presented participants with more or fewer good or poor arguments. At the theoretical level, it is not surprising that inducing participants to devote much versus little processing effort would influence the relative effectiveness of cues designed to work better with much or little processing effort. However, researchers at the time considered it surprising that argument quality and argument quantity would function in these two ways, respectively. Thus, the finding that argument quality mattered more when participants devoted more processing effort, and that argument quantity mattered more when participants devoted less processing effort, counted as a surprising finding. But the surprise was at the methodological level rather than at the theoretical level.

A third possible combination is for both the theory and the auxiliary assumptions to be surprising. This combination rarely happens in psychology, and no cases come immediately to mind. If it were to happen, perhaps reviewers would think that the author pulled a fast one on them. And the fourth combination is for both the theory and the hypothesis to be obvious, in which case the manuscript probably would not be accepted by a top journal unless the reviewers felt that they had an interest in having the manuscript published, perhaps because the manuscript cited their work in a positive way.

In conclusion, we should be asking why a surprising finding is surprising. If the surprise results from the prior implausibility of the theory, I would agree that the surprising finding likely constitutes a real contribution to science. However, if the surprise is of the “you got that to work!” variety, where the surprise resides in the auxiliary assumptions, I am not impressed. Researchers in other sciences, such as physics, understand well the difference between different kinds of surprise. It is high time for psychology researchers to do so too.
